# Barriers and enablers of HPV vaccination among caregivers of school-age girls in resource-limited regions of Western Sichuan, China: a mixed-methods study

**DOI:** 10.3389/fpubh.2025.1618935

**Published:** 2025-08-20

**Authors:** Qiwen Zhang, Lu Zhang, Zubo Huang, Yue Zeng, Yueshan Wang, Jieru Peng, Yan Kou, Wei Huang, Gang Zhu, Wencheng Long, Yao Dong, Chunxia Yang

**Affiliations:** ^1^Department of Epidemiology and Biostatistics, West China School of Public Health and West China Fourth Hospital, Sichuan University, Chengdu, China; ^2^Sub-Health Department, Sichuan Integrative Medicine Hospital, Chengdu, China; ^3^Songpan County Center for Disease Control and Prevention, Songpan, China; ^4^Mianning County Maternal and Child Health Hospital, Mianning, China

**Keywords:** HPV vaccination, vaccination willingness, immunization policy, mixed methods, cervical cancer prevention

## Abstract

**Background:**

The HPV infection rate in Sichuan exceeds the national average. The provincial government has initiated vaccination programs, with a particular focus on impoverished areas such as the Ganzi, Aba, and Liangshan prefectures, where both the HPV disease burden is elevated and vaccination uptake remains suboptimal. This study aimed to investigate HPV vaccination among adolescent girls in these prefectures, evaluate caregivers’ knowledge and attitudes, identify factors influencing their willingness to vaccinate their daughters, and examine existing policies to provide evidence-based recommendations for improving vaccination strategies.

**Methods:**

A cross-sectional study was conducted in Ganzi, Aba, and Liangshan prefectures from September 2023 to May 2024, integrating both quantitative and qualitative methods. At least 2,034 caregivers and 75 participants should be recruited for the quantitative and qualitative studies, respectively. Questionnaires were used to assess caregivers’ willingness to vaccinate girls under the age of 15. Quantitative analyses included Chi-square tests, Mann–Whitney U tests, and logistic regression analyses. Additionally, focus group discussions and face-to-face interviews with vaccination personnel and caregivers explored vaccination coverage and barriers, with qualitative data analyzed via thematic framework analysis.

**Results:**

A total of 2,397 caregivers completed valid questionnaires, with 59.28% having heard of HPV and 69.63% being aware of the HPV vaccine. Although 92.12% expressed willingness to vaccinate their girls against HPV, the actual vaccination rate was only 6.59%. Reluctance to vaccinate was primarily due to a lack of knowledge about the vaccine (30.69%), concern about potential side effects (28.04%), and uncertainty about the vaccine’s effectiveness (18.5%). Regression analysis showed that caregivers who were mothers, willing to pay for HPV vaccination, personally willing to receive the HPV vaccine, and had never refused a vaccine were more likely to vaccinate girls against HPV (all *p* < 0.05). Qualitative findings further identified economic constraints, insufficient vaccine supply, and limited public awareness as major barriers to HPV vaccination.

**Conclusion:**

Caregivers hold positive attitudes toward HPV vaccination, but the coverage rate among girls remains low in Ganzi, Aba, and Liangshan prefectures. Ensuring vaccine supply, reducing costs, improving caregivers’ knowledge, and increasing vaccination accessibility could significantly enhance vaccination rates in resource-limited regions of western China.

## 1 Introduction

Human papillomavirus (HPV) infection is the most common sexually transmitted infection worldwide, and persistent infection by the high-risk HPV types increases the risk of progression to cervical cancer (1). While the incidence of cancers attributed to HPV infection in China is lower than the global average, the absolute number of cases is significant, accounting for approximately 20% of the world’s total (2). Furthermore, there is a rising trend in both morbidity and mortality (2).

Significant disparities in HPV infection prevalence and healthcare access exist within China, particularly in its western provinces. As one of the typically impoverished provinces in western China, Sichuan Province ranks low in health resources, with significant disparities in development (3). The reported prevalence of HPV infection in Sichuan is 23.84% (4), which is higher than the national average (15.5–18.7%) (5, 6). Crucially, this disparity is exacerbated within the province itself. Health resources are heavily concentrated in urban centers, while rural areas, especially the Ganzi-Aba-Liangshan (GAL) prefectures in western Sichuan, demonstrate markedly slower progress in health service development (7). The GAL prefectures are remote, mountainous regions inhabited by ethnic minorities, with less developed economies. The number of medical institutions, health professionals, and hospital beds was much lower than in other parts of Sichuan (7), making these areas typical examples of regions with poor access to healthcare in western China. Consequently, the burden of HPV-related disease in the GAL prefectures is likely to be substantial. This confluence of factors, a high-prevalence province and severe healthcare access barriers, makes the GAL prefectures a critical high-priority area for targeted HPV vaccination intervention (8).

In 2018, the World Health Organization (WHO) issued a global call to action aimed at eliminating cervical cancer by enhancing vaccination, screening, and treatment efforts. They recommended routine HPV vaccination for girls aged 9 to 14 years, ideally before they become sexually active (9). China has accorded high priority to HPV vaccination promotion (10). While HPV vaccines have not yet been incorporated into the National Immunization Program (NIP), provincial authorities are encouraged to develop tailored vaccination strategies based on local fiscal capacity and immunization needs, such as financial subsidies and free provision of domestically produced bivalent HPV vaccines. In response to this initiative, Chengdu, the capital of Sichuan Province, conducted a survey in 2021 to assess the knowledge, attitudes, and practices (KAP) as well as willingness to pay for HPV vaccination among parents of schoolgirls aged 13–14. The findings from this survey led to the development of a city immunization program (CIP) strategy, which included a subsidy of RMB 600 per person and allowed parents to choose the HPV vaccine voluntarily. Consequently, within just 2 months of the CIP’s implementation, the HPV vaccination coverage rate among female students aged 13 to 14 surged to an impressive 90%. This city immunization program serves as a strong role model for other regions of Sichuan and other provinces in western China. Currently, the Sichuan government is working to expand HPV vaccination programs for adolescent girls across the province, including impoverished areas such as GAL. It is worth noting that the GAL prefectures are economically underdeveloped, with a per capita GDP of RMB 46,159 to 61,067 (approximately US$6,367 to 8,423) in 2023. In contrast, the complete cost of a three-dose HPV vaccination ranged from RMB 1,740 to 3,954 (approximately US$254 to $576) (11). The high cost of vaccination may pose a substantial barrier to immunization uptake in these regions (12, 13), highlighting the need for government policies that promote health equity. However, due to the lack of research on HPV vaccination in these regions, health awareness, willingness to receive the vaccine, vaccine selection, and their influencing factors remain unclear. This data gap has hindered the timely implementation of the HPV vaccination program across the province.

Existing literature indicates that HPV vaccination coverage among adolescents is strongly influenced by their caregivers’ willingness, positioning it as a critical focus for vaccine promotion efforts (14, 15). However, vaccination uptake is also shaped by broader governmental and societal factors, including policy support, vaccine accessibility, and economic barriers, which remain understudied in the GAL prefectures. Therefore, this study employed a mixed-methods design. The primary analyses conducted by quantitative methods aimed to investigate the current status of HPV vaccination among adolescent girls, along with their caregivers’ knowledge, attitudes, and practices (KAP), and identify factors influencing their willingness to vaccinate their daughters. Qualitative interviews were conducted with caregivers and vaccination personnel to further explore relevant vaccination policies and barriers in the GAL prefectures. Quantitative findings were further supplemented through qualitative interviews. This integrated approach aims to inform the development of tailored and feasible HPV vaccination strategies for the GAL regions, thereby contributing to the prevention and control of HPV-related diseases.

## 2 Materials and methods

### 2.1 Study design

**Figure 1:**
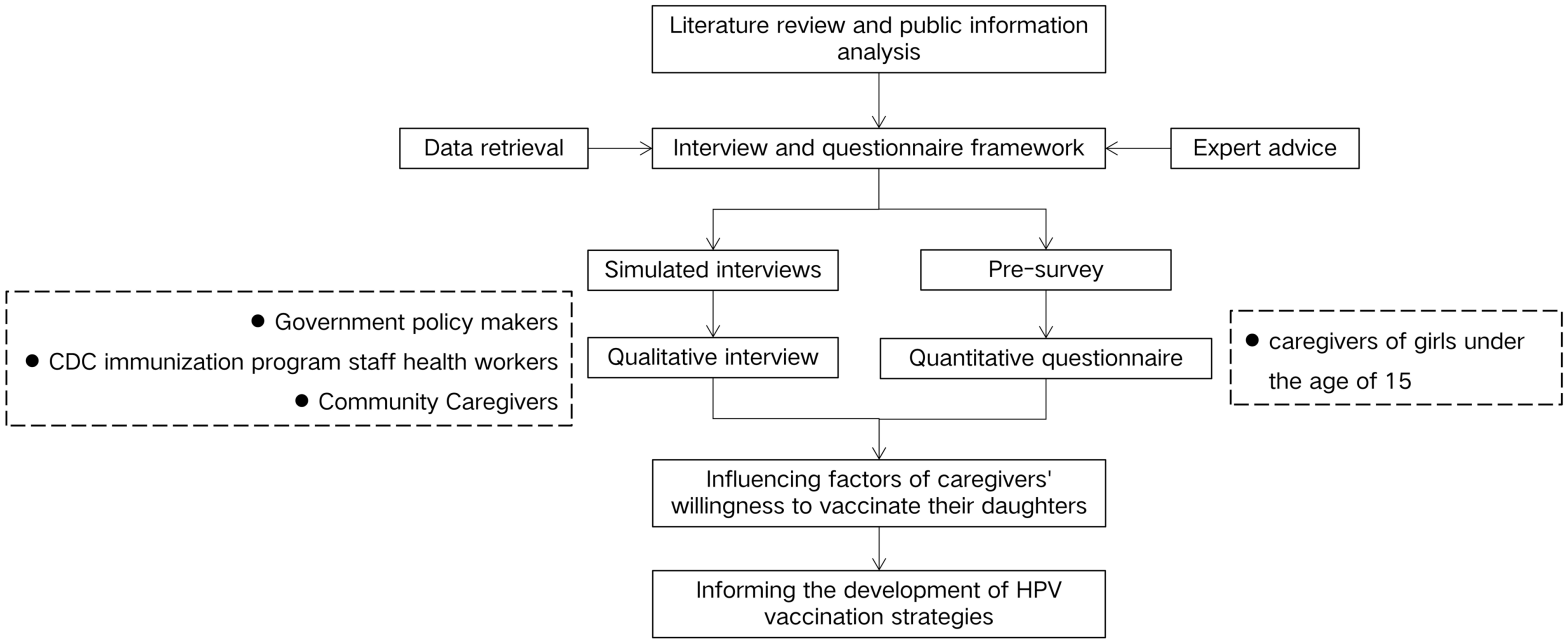
The flowchart of the study design.

The research, conducted from September 2023 to May 2024 in the GAL prefectures of Sichuan Province, employed a cross-sectional design incorporating both quantitative and qualitative methods. A quantitative study was conducted to evaluate the knowledge and attitudes of caregivers of primary and secondary school girls in the GAL areas regarding HPV vaccines, utilizing questionnaires for data collection. Additionally, a qualitative study involving focus group discussions (FGDs) and face-to-face interviews was conducted to assess local HPV vaccination coverage, explore factors influencing vaccination uptake, and provide deeper insights into caregivers’ perspectives that could not be fully captured by quantitative findings alone, with the aim of informing measures and policy recommendations to improve vaccination rates., The results from both methodologies were analyzed to identify the factors influencing HPV vaccination among adolescent girls in the GAL areas. The flowchart of the study design is shown in Figure 1.

### 2.2 Sampling

#### 2.2.1 Quantitative sample

In the quantitative questionnaire survey, stratified cluster sampling was used to select at least one primary school and one middle school from both urban and rural areas in Ganzi, Aba, and Liangshan prefectures, respectively. Caregivers of female students from these schools were selected as the study population. Inclusion criteria: (1) individuals who could independently complete the survey and had no severe mental disorders; (2) caregivers of girls under the age of 15; (3) those who agreed to participate in the survey.

The sample size was estimated based on the primary outcome (vaccination willingness). The calculation formula is as follows:

$n=\frac{u_{\alpha /2}^{2}\pi (1-\pi )}{\delta^{2}}(\delta$=0.1π, *α* = 0.05, $u_{\alpha /2}$=1.96).

No research has been conducted on HPV vaccination willingness in GAL areas, so the estimation was based on data from Chengdu, where the willingness to vaccinate their children among parents of middle school students was 47.97% (16). Using 80% of this figure as a benchmark, the estimated vaccination willingness in GAL areas was calculated to be 38.38%, leading to an estimated sample size of *n* = 616. To ensure sufficient sample size and survey quality, the sample size was increased by at least 10%, totaling approximately 678 participants per region. Consequently, at least 2,034 participants should be included across the three regions.

#### 2.2.2 Qualitative sample

Targeted sampling was used to recruit participants for the qualitative research from Ganzi, Aba, and Liangshan prefectures. The participants included: (1) government policymakers; (2) leader and vaccination-related personnel from CDC as well as Maternal and Child Health Hospital; (3) immunization staff from community health service centers or township health centers; and (4) caregivers of primary and secondary school female students aged 9–14. Inclusion criteria: (1) individuals who could independently complete the survey and had no severe mental disorders; and (2) those who agreed to participate in the survey.

Based on the feasibility and necessity of the research, and following the principle of information saturation, a total of 75 participants were included in the qualitative interviews, with 25 participants from each of the three regions. In each region, 10 vaccination-related personnel were selected to participate in focus group discussions or face-to-face interviews. These participants included leaders and staff members from the Health Commission and CDC, immunization personnel from community or township health centers, and representatives from Maternal and Child Health Hospital. Additionally, 15 caregivers of primary and secondary school students aged 9–14 were recruited from each region for face-to-face interviews. Participants received small gifts as compensation.

### 2.3 Measurements tools

#### 2.3.1 Quantitative questionnaire development

The questionnaire (see Appendix A) for caregivers, consisting of 36 questions, was developed by the research team based on a literature review, refined through expert consultation, and adjusted based on the pre-survey. Reliability analysis indicated acceptable internal consistency for the dimensions of vaccination willingness (Cronbach’s *α* = 0.611) and knowledge level (Cronbach’s α = 0.723). The finalized questionnaire included the following sections: (1) demographic characteristics, such as age, education level, occupation, and marital status, etc.; (2) caregivers’ knowledge of HPV and HPV vaccine; (3) willingness to vaccinate and reasons for accepting or refusing vaccination; (4) caregivers’ practices concerning vaccination for themselves and their daughters with various vaccines; (5) other facilitating factors for caregivers to vaccinate their daughters, including policy-related elements.

Knowledge of HPV and HPV vaccine was assessed through a series of related questions. Awareness of HPV was measured with the question “Have you heard of Human papillomavirus?.” Additional questions evaluated caregivers’ knowledge levels on HPV and the HPV vaccine, including the diseases the vaccine can prevent and the optimal age for vaccination. All the questions were adapted from previous research (17, 18). Each correct answer regarding HPV and the HPV vaccine earned 1 point, while incorrect answers or responses indicating “unknown” received 0 points. The total knowledge score could range from 0 to 6. Respondents were then divided into three groups based on their total knowledge scores: low knowledge (score = 0–2), medium knowledge (score = 3–4) and high knowledge (score = 5–6).

The question “If given the opportunity, would you be willing to vaccinate your children with HPV vaccine?” assessed caregivers’ willingness to vaccinate their daughters against HPV. Caregivers who answered ‘No’ were further asked to select their primary reason for not accepting the HPV vaccine from a multiple-choice question with 13 specified options. Caregivers who answered ‘Yes’ were asked to select their main reasons for willingness to vaccinate from a multiple-choice question with 7 specified options.

Caregivers’ vaccination practices for both themselves and their daughters were assessed through a series of questions, including their willingness to receive all scheduled and recommended vaccines for themselves and their children, any history of delaying or hesitating to vaccinate, any history of vaccine refusal, and whether they had opted for self-funded vaccines for their children. Other facilitators influencing caregivers’ willingness to vaccinate their daughters were assessed through a series of questions regarding access to HPV-related information, selection of vaccination sites, and governmental subsidy policies.

#### 2.3.2 Qualitative interview guide

Qualitative data were collected from participants in the GAL areas through FGDs or face-to-face interviews. The interview guides were developed by the research team based on comprehensive literature reviews and expert consultations, refined through guideline workshops, and further optimized through simulated interviews to improve questioning strategies and ensure smooth implementation of formal interviews.

The FGDs with vaccination-related personnel and health department leaders were conducted following a semi-structured interview guide (see Appendix B). The interviews primarily aimed to collect the following information: (1) current status of HPV vaccination among adolescent girls and local relevant policies; (2) factors influencing HPV vaccination, especially policies like payment subsidies, community organization, and vaccine supply; and (3) challenges and solutions for promoting HPV vaccination in areas with limited healthcare resources. Additionally, a separate semi-structured interview guide (see Appendix C) was used to conduct face-to-face interviews with caregivers of girls aged 9–14 in the region. The following information was collected: (1) the HPV vaccination status of eligible girls around the interviewed caregivers; (2) caregivers’ knowledge about HPV and HPV vaccine; and (3) factors influencing HPV vaccination.

### 2.4 Data collection

#### 2.4.1 Quantitative survey

The study was conducted from September 2023 to April 2024. In collaboration with the local CDC, the purpose, content, and estimated time to complete the questionnaire were explained to the relevant school departments. After receiving consent and support from the school authorities, paper questionnaires were distributed to female students in their classes by their head teachers. Female students took the questionnaire home with written instructions for their caregivers. Caregivers were asked to complete the questionnaire independently without consulting others or external resources. The completed questionnaires were then brought back to school to the head teachers the following day. If any missing values were found, the teacher would ask the student to take the questionnaire back home for their caregivers to fill in the missing parts before resubmitting it, along with more detailed instructions and guidance on how to complete it. Upon collection, researchers reviewed the questionnaires for missing responses, incorrect entries, and logical errors. Questionnaires with incomplete answers, logical errors or all identical answers were marked as invalid.

#### 2.4.2 Qualitative interview

In each region, FGD was held with vaccination-related personnel and leaders from the health department. The discussion took place in conference room provided by CDC or Health Commission. Each FGD was led by an experienced female researcher specialized in HPV-related programs. Two research assistants were present to take notes and assist with logistics. Interviews lasted an average of 45–60 min and followed an interview guide. FGDs were audio-recorded with permission. After the FGDs, in-person interviews were conducted with individuals requiring further discussions. This included leaders from the Health Commission, representatives from the Maternal and Child Health Centers, and heads of the immunization planning section at the CDC. The goal was to enhance and refine the findings.

Face-to-face interviews were held with 15 caregivers of girls aged 9–14 years in each region. Three trained interviewers conducted the interviews, having received standardized training prior to the study to ensure consistency in questioning techniques. Interviews were held in school conference rooms, each lasting 25 to 40 min depending on participants’ HPV knowledge and exposure to HPV vaccine information. All interviews were performed in the Sichuan dialect, as participants were caregivers from the GAL areas. With participants’ consent, interviews were audio-recorded.

Upon concluding the interviews, the interviewers transcribed the recordings into text, and two researchers examined the transcriptions against the recordings to prevent omissions and inaccuracies. The transcribed data were then translated, organized, and categorized to form preliminary interpretations, which were subsequently validated and corrected by referring back to the original interview recordings. Qualitative data from the transcripts and translations were coded and categorized according to inductive thematic analysis using NVivo 12 software (19). Conceptual saturation was reached when no new themes or subthemes were generated from the data.

### 2.5 Statistical analysis

EpiData 3.1 was applied for the data entry of the questionnaires. All statistical analyses were performed using IBM SPSS Statistics 29.0. Descriptive statistics were used to analyze the sociodemographic characteristics, the knowledge about HPV and the HPV vaccine, HPV vaccine knowledge scores, and attitudes toward HPV vaccination. The data normality was evaluated using Kolmogorov–Smirnov tests. Results were presented as frequencies and proportions for categorical variables, median (interquartile range) for non-normally distributed continuous variables, and mean ± standard deviation (SD) for normally distributed continuous variables. Univariable and multivariable analyses were conducted on caregivers’ willingness to vaccinate their girls with the HPV vaccine. The Chi-square test or Fisher’s exact test (if the expected frequency was <5%) was used for group comparisons of categorical variables, and HPV vaccine knowledge scores were analyzed using the Mann–Whitney U rank-sum test. Variables showing significant differences in the univariable analysis were included in a multivariable binary logistic regression analysis. The two-sided *p* < 0.05 indicated statistical significance.

## 3 Results

### 3.1 Quantitative survey results

#### 3.1.1 Participants characteristics

**Figure 2:**
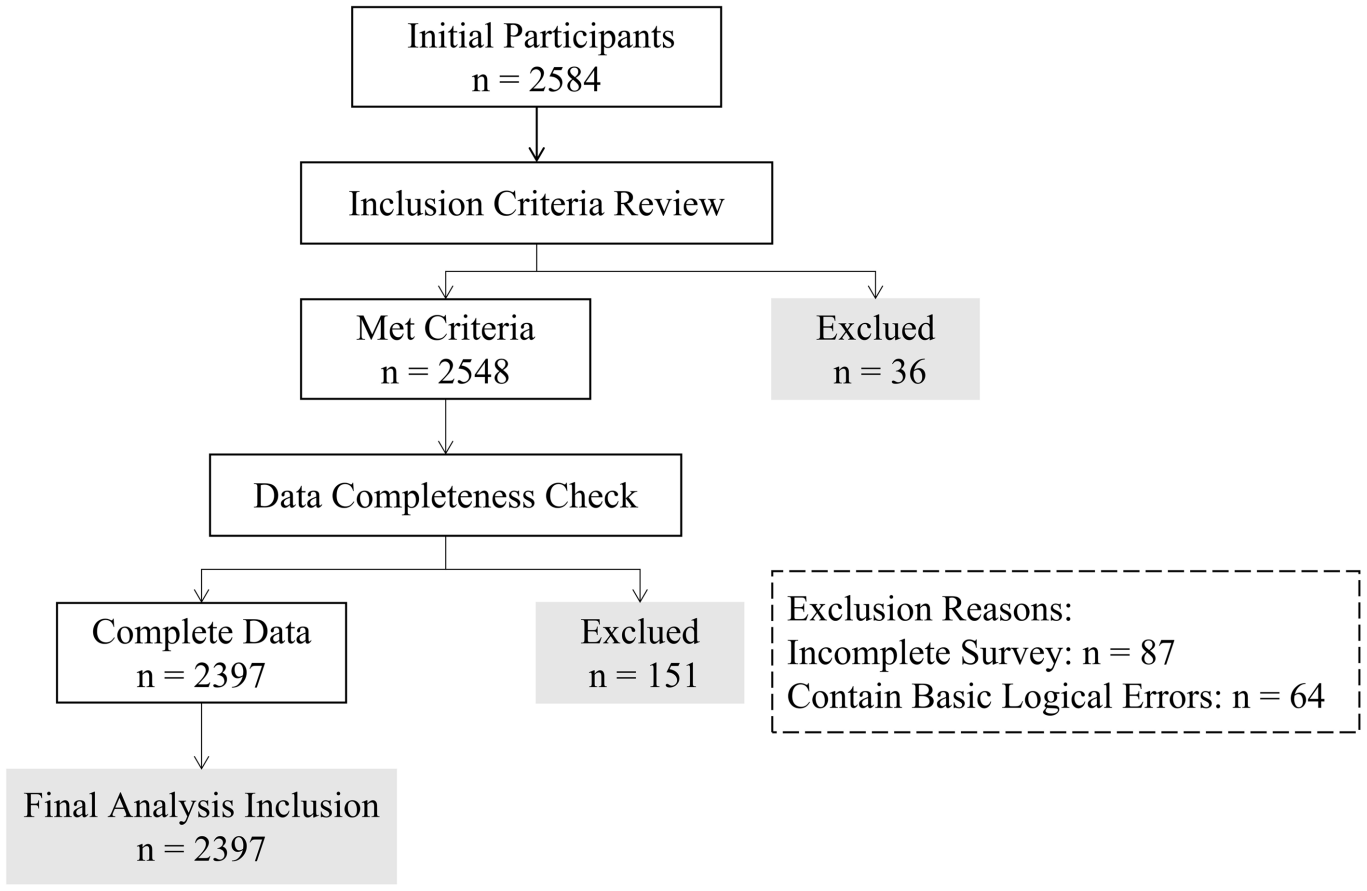
A schematic diagram of the selection of participants for data.

As shown in Figures 2, a total of 2,584 caregivers of primary and secondary school girls were enrolled to participate, and 2,397 participants (92.76%) who completed the survey were included in the analysis.

There are 971 (40.51%) participants from Ganzi Prefecture, 775 (32.33%) from Aba Prefecture, and 651 (27.16%) from Liangshan Prefecture. Most participants were mothers of girls (68.25%), married (92.03%), rural residents (53.73%), ethnic minorities (58.03%), with an education level of junior high school and below (69.63%). 35.17% of participants work as homemakers. Most girls were not the only child in their family (87.40%) and attended primary and secondary schools in urban areas (63.83%). All demographic characteristics are presented in Table 1.

**Table 1:** Demographic characteristics of participants (*n* = 2,397).

Characteristics	N	%	Characteristics	N	%
Region			Educational level		
Ganzi Prefecture	971	40.51	No Formal Education	215	8.97
Aba Prefecture	775	32.33	Primary School	644	26.87
Liangshan Prefecture	651	27.16	Junior High School	810	33.79
Relationship with the girl			Senior High School	277	11.56
Mother	1,648	68.75	College and Above	451	18.82
Father	601	25.07	Occupation		
Others	148	6.17	Staff/Officer	370	15.44
Marital Status			Factory Worker/Industrial Worker	281	11.72
Unmarried	19	0.79	Service Worker	84	3.50
Married	2,206	92.03	Business personnel/Professional	300	12.52
Divorced	147	6.13	Homemaker/Housewife	843	35.17
Widowed	25	1.04	Others	519	21.65
Residence area			Is the girl the only child		
Rural	1,288	53.73	Yes	302	12.60
Urban	1,109	46.27	No	2095	87.40
Ethnicity			The girl’s school		
Han	1,006	41.97	Rural primary school	510	21.28
Tibetan	895	37.34	Rural junior high school	357	14.89
Yi	245	10.22	Urban primary school	863	36.00
Qiang	91	3.80	Urban junior high school	667	27.83
Others	160	6.68			

#### 3.1.2 The knowledge of caregivers about HPV and the HPV vaccines

Among the 2,397 caregivers, most had heard of Human Papillomavirus (59.28%) and HPV vaccine (69.63%) before the survey. The majority of caregivers know that the HPV vaccine can prevent cervical cancer (69.50%), while awareness that the best age for receiving the HPV vaccine (43.01%) was relatively low. Moreover, only a small percentage of caregivers were aware that the HPV vaccine can prevent anal cancer (14.14%) and genital warts (22.99%). See Table 2 for details.

**Table 2:** Caregivers’ knowledge of HPV and HPV vaccines (*n* = 2,397).

Variables	N	%	95%*CI*
Have heard of HPV	1,421	59.28	(57.30,61.23)
Have heard of the HPV vaccine	1,669	69.63	(67.76,71.44)
HPV vaccine can prevent cervical cancer	1,666	69.50	(67.63,71.31)
HPV vaccine can prevent anal cancer	339	14.14	(12.80,15.60)
HPV vaccine can prevent genital warts	551	22.99	(21.35,24.71)
The best age for receiving the HPV vaccine	1,031	43.01	(41.04,45.00)

CI, Confidence interval; HPV, Human papillomavirus.

Median and interquartile range (IQR) of HPV and HPV vaccine-related knowledge scores were 3 (2, 4) out of a total score of 6. Most caregivers (52.07%) had a medium level of knowledge about HPV and HPV vaccines, while only 266 caregivers (11.10%) were considered to have a high level of knowledge.

#### 3.1.3 Caregivers’ willingness, attitudes and practices toward HPV vaccination

**Figure 3:**
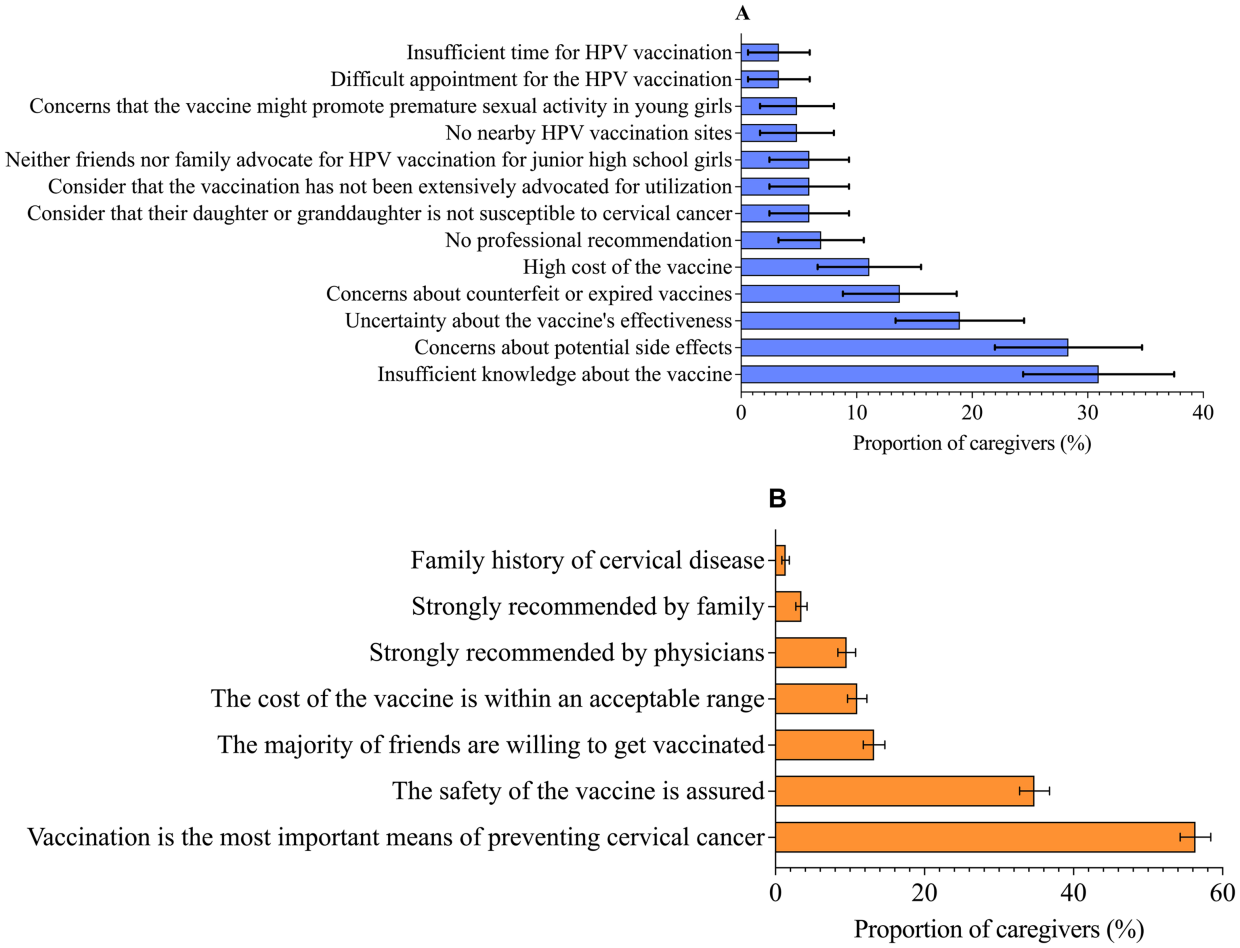
Reasons for caregivers unwilling or willing to vaccinate adolescent daughters against HPV. **(A)** Reasons for caregivers unwilling to vaccinate adolescent daughters against HPV. **(B)** Reasons for caregivers willing to vaccinate adolescent daughters against HPV. Error bars represent 95% *CI*.

92.37% of caregivers expressed willingness to vaccinate themselves against HPV, while 92.12% were willing to vaccinate their adolescent daughters. Only 7.88% of caregivers refused to vaccinate their daughters against HPV, with the common reasons including “Insufficient knowledge about the vaccine” (30.69%), “Concerns about potential side effects” (28.04%), and “Uncertainty about the vaccine’s effectiveness” (18.52%). Among those willing to vaccinate, most (56.39%) believed that “Vaccination is the most important means to prevent cervical cancer,” and 34.74% of caregivers felt that “The safety of the vaccine is assured.” See Table 3 and Figure 3 for details.

**Table 3:** Caregivers’ willingness, attitudes and practices toward vaccination (*n* = 2,397).

Variables	N	%
Willingness toward vaccination		
Personal willingness to get the HPV vaccine		
Yes	2,214	92.37
No	183	7.63
Willingness to vaccinate adolescent daughters against HPV		
Yes	2,208	92.12
No	189	7.88
Willingness to pay for HPV vaccination (yuan)		
No	425	17.73
≤ 200	916	38.21
200–500	834	34.79
>500	222	9.26
Willingness to get all scheduled and recommended vaccines for themselves		
Yes	2026	84.52
No	371	15.48
Willingness to get all scheduled and recommended vaccines for their children		
Yes	2016	84.11
No	381	15.89
Vaccination hesitancy		
History of hesitating or delaying to vaccinate themselves		
Yes	898	37.46
No	1,499	62.54
History of hesitating or delaying to vaccinate their children		
Yes	617	25.74
No	1780	74.26
History of vaccine refusal for themselves		
Yes	271	11.31
No	2,126	88.69
History of vaccine refusal for their children		
Yes	194	8.09
No	2,203	91.91
Vaccination practices		
History of getting self-paid vaccines for their children		
Yes	1,490	62.16
No	907	37.84
History of getting HPV vaccine for their children		
Yes	158	6.59
No	2,239	93.41

HPV, Human papillomavirus.

17.73% of caregivers were unwilling to pay out of pocket for the HPV vaccine, 38.21% were only willing to pay 200 RMB or less, while only 9.26% of caregivers could afford more than 500 RMB. Despite a majority of caregivers expressed a willingness to receive all scheduled and nationally recommended vaccines for themselves (84.52%) and their children (84.11%), vaccine hesitancy remained a concern. 37.46 and 25.74% of caregivers reported that they had intentionally postponed or hesitated to receive vaccines for themselves or their children, and 11.31 and 8.09% of caregivers had refused vaccination for themselves and their children. Additionally, regarding vaccination practices, 62.16% of caregivers reported that they had vaccinated their children at their own expense, while only 6.59% had vaccinated their daughters against HPV. See Table 3 for details.

**Figure 4:**
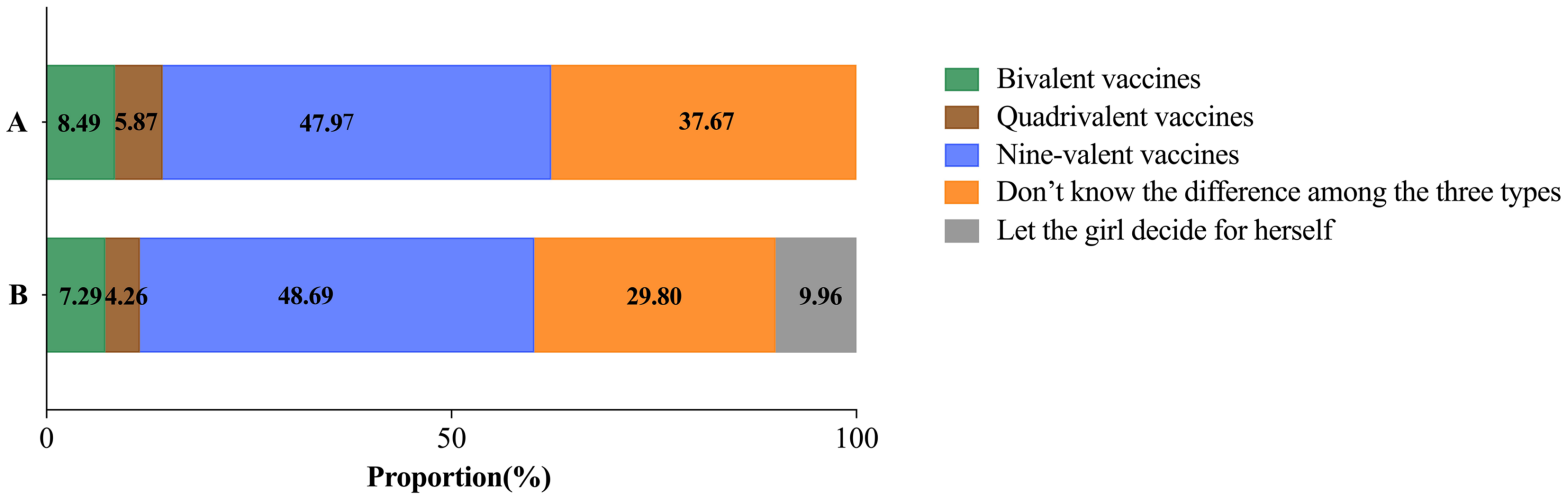
The proportion of caregivers willing to receive different types of HPV vaccines for themselves and their adolescent daughters. **(A)** Preferred HPV vaccine type among caregivers willing to vaccinate themselves. **(B)** Preferred HPV vaccine type among caregivers willing to vaccinate their adolescent daughters.

The majority of caregivers (48.69%) preferred to wait for the nine-valent HPV vaccine while only a minority of caregivers chose to get either bivalent or quadrivalent HPV vaccine for their children (11.55%) as soon as possible. Furthermore, 9.96% of caregivers expressed a willingness to let their girls choose their preferred type of vaccine independently. However, many caregivers lacked understanding of the differences among the three types (29.80%) and expressed hesitation regarding their decisions. See Figure 4 for details.

#### 3.1.4 Factors associated with caregivers’ willingness to vaccinate adolescent daughters with HPV vaccine

The Chi-square test results indicated that relationship with the girl, marital status of caregivers, awareness of HPV and the HPV vaccine, knowledge level and scores, willingness to cover the cost of vaccination, personal willingness to get the HPV vaccine, willingness to get all scheduled and recommended vaccines for themselves and their children, as well as any history of vaccine refusal for themselves or their children affected the willingness of caregivers (all *p* < 0.05). Additionally, the Mann–Whitney U test revealed a statistically significant difference in knowledge scores between those willing and unwilling to vaccinate their daughters (*p* < 0.001). Caregivers with higher levels of knowledge were more likely to vaccinate their daughters against HPV. See Table 4 for details.

**Table 4:** Caregivers’ willingness toward HPV vaccination for their adolescent daughters.

Variables	*N*	Caregivers’ willingness to vaccinate adolescent daughters against HPV; N(%) / *M*(*P_25_*, P*_75_*)		*Z/* $\chi^{2}$	*p*
		Willingness	Unwillingness		
Sociodemographic characteristics					
Relationship with the girl					
Mother	1,648	1,544(93.69)	104(6.31)	21.134	**<0.001**
Father	601	538(89.52)	63(10.48)		
Others	148	126(85.14)	22(14.86)		
Total	2,397	2,208	189		
Is the girl the only child					
Yes	302	282(93.38)	20(6.62)	0.758	0.384
No	2095	1926(91.93)	169(8.07)		
Total	2,397	2,208	189		
The girl’s school					
Rural primary school	510	466(91.37)	44(8.63)	6.594	0.086
Rural junior high school	357	319(89.36)	38(10.64)		
Urban primary school	863	798(92.47)	65(7.53)		
Urban junior high school	667	625(93.70)	42(6.30)		
Total	2,397	2,208	189		
Residence area					
Rural	1,288	1,181(91.69)	107(8.31)	0.685	0.408
Urban	1,109	1,027(92.61)	82(7.39)		
Total	2,397	2,208	189		
Region					
Ganzi Prefecture	971	892(91.86)	79(8.14)	5.950	0.051
Aba Prefecture	775	703(90.71)	72(9.29)		
Liangshan Prefecture	651	613(94.16)	38(5.84)		
Total	2,397	2,208	189		
Ethnicity					
Han	1,006	935(92.94)	71(7.06)	7.994	0.092
Tibetan	895	809(90.39)	86(9.61)		
Yi	245	233(95.10)	12(4.90)		
Qiang	91	85(93.41)	6(6.59)		
Others	160	146(91.25)	14(8.75)		
Total	2,397	2,208	189		
Educational level					
No Formal Education	215	200(93.02)	15(6.98)	6.492	0.165
Primary School	644	590(91.61)	54(8.39)		
Junior High School	810	734(90.62)	76(9.38)		
Senior High School	277	259(93.502)	18(6.498)		
Associate Degree and Above	451	425(94.24)	26(5.76)		
Total	2,397	2,208	189		
Occupation					
Staff / Officer	370	347(93.78)	23(6.22)	4.770	0.445
Factory Worker/Industrial Worker	281	261(92.88)	20(7.12)		
Service Worker	84	76(90.48)	8(9.52)		
Business personnel/Professional	300	277(92.33)	23(7.67)		
Homemaker/Housewife	843	779(92.41)	64(7.59)		
Others	519	468(90.17)	51(9.83)		
Total	2,397	2,208	189		
Marital Status					
Unmarried	19	15(78.95)	4(21.05)	9.583	**0.022**
Married	2,206	2042(92.57)	164(7.43)		
Divorced	147	129(87.76)	18(12.24)		
Widowed	25	22(88.00)	3(12.00)		
Total	2,397	2,208	189		
Knowledge Status					
Have heard of HPV					
Yes	1,421	1,339(94.23)	82(5.77)	21.479	**<0.001**
No	976	869(89.04)	107(10.96)		
Total	2,397	2,208	189		
Have heard of the HPV vaccine					
Yes	1,669	1,565(93.77)	104(6.23)	20.688	**<0.001**
No	728	643(88.32)	85(11.68)		
Total	2,397	2,208	189		
Knowledge level*					
Low knowledge	883	788(89.24)	95(10.76)	16.042	**<0.001**
Medium knowledge	1,248	1,169(93.67)	79(6.33)		
High knowledge	266	251(94.36)	15(5.64)		
Score	--	3(2,4)	2(1,3)	−4.691	**<0.001**
Total	2,397	2,208	189		
Willingness toward vaccination					
Personal willingness to receive the HPV vaccine					
Yes	2,214	2,134(96.39)	80(3.61)	728.497	**<0.001**
No	183	74(40.44)	109(59.56)		
Total	2,397	2,208	189		
Willingness to pay for HPV vaccination (yuan)					
No	425	351(82.59)	74(17.41)	69.585	**<0.001**
≤ 200	916	854(93.23)	62(6.77)		
200–500	834	786(94.24)	48(5.76)		
>500	222	217(97.75)	5(2.25)		
Total	2,397	2,208	189		
Willingness receive all scheduled and recommended vaccines for themselves					
Yes	2026	1881(92.84)	145(7.16)	9.549	**0.002**
No	371	327(88.14)	44(11.86)		
Total	2,397	2,208	189		
Willingness receive all scheduled and recommended vaccines for their children					
Yes	2016	1875(93.01)	141(6.99)	13.857	**<0.001**
No	381	333(87.40)	48(12.60)		
Total	2,397	2,208	189		
Vaccination hesitancy					
History of hesitating or delaying to vaccinate themselves					
Yes	898	826(91.98)	72(8.02)	0.035	0.852
No	1,499	1,382(92.19)	117(7.81)		
Total	2,397	2,208	189		
History of hesitating or delaying to vaccinate their children					
Yes	617	569(92.22)	48(7.78)	0.013	0.910
No	1780	1,639(92.08)	141(7.92)		
Total	2,397	2,208	189		
History of vaccine refusal for themselves					
Yes	271	226(83.39)	45(16.61)	31.990	**<0.001**
No	2,126	1982(93.23)	144(6.77)		
Total	2,397	2,208	189		
History of vaccine refusal for their children					
Yes	194	163(84.02)	31(15.98)	19.042	**<0.001**
No	2,203	2045(92.83)	158(7.17)		
Total	2,397	2,208	189		
Vaccination practices					
History of receiving self-paid vaccines for their children					
Yes	1,490	1,385(92.95)	105(7.05)	3.806	0.051
No	907	823(90.74)	84(9.26)		
Total	2,397	2,208	189		
History of receiving HPV vaccine for their children					
Yes	158	147(93.04)	11(6.96)	0.198	0.656
No	2,239	2061(92.05)	178(7.95)		
Total	2,397	2,208	189		

*Knowledge level: low knowledge (score = 0–2), medium knowledge (score = 3–4) and high knowledge (score = 5–6). HPV, Human papillomavirus. Bold: results significant (*p* < 0.05).

Binary logistic regression was performed using caregivers’ willingness to vaccinate their adolescent daughters against HPV as the dependent variable (0 = unwillingness, 1 = willingness). The independent variables were 12 factors that were statistically significant (*p* < 0.05) in the univariable analysis. The factors significantly associated with caregivers’ willingness to vaccinate their adolescent daughters against HPV were the caregiver’s relationship with the girl, willingness to cover part of the vaccination costs, personal willingness to receive the HPV vaccine, and whether they had ever refused vaccination before (all *p* < 0.05). The regression model demonstrated a good fit ($\chi^{2}$=6.271, *p* = 0.393), and there was no significant multicollinearity among the variables (VIF = 1.044–2.117). Mothers (*OR* = 2.74, 95% *CI*: 1.48–5.05) and caregivers personally willing to receive the HPV vaccine (*OR* = 35.18, 95% *CI*: 23.92–51.74) were more likely to have their daughters vaccinated. Conversely, caregivers with a history of vaccination refusal (*OR* = 0.51, 95% *CI*: 0.32–0.81) demonstrated greater reluctance to vaccinate their daughters. Higher caregiver willingness to cover HPV vaccination costs also significantly predicted increased vaccination uptake. Odds ratios (ORs) varied across cost tiers: ≤200 RMB (*OR* = 2.17, 95% *CI*: 1.39–3.40), 200–500 RMB (*OR* = 1.94, 95% *CI*: 1.21–3.11), and >500 RMB (*OR* = 5.02, 95% *CI*: 1.81–13.92), collectively suggesting a positive association between willingness to pay and the likelihood of daughter vaccination. See Table 5 for details.

**Table 5:** Binary logistic regression analysis of factors associated with vaccination willingness.

Factors	*β*	*SE*	Wald $\chi^{2}$	*p*	*OR* (95%*CI*)
Constant	−1.595	0.355	20.166	**0.005**	0.20
Relationship to the girl					
Other	reference				
Mother	1.006	0.313	10.329	**0.001**	2.74(1.48,5.05)
Father	0.632	0.333	3.608	0.058	1.88 (0.98,3.61)
Willingness to pay for vaccination (yuan)					
No	reference				
≤ 200	0.775	0.229	11.483	**<0.001**	2.17 (1.39,3.40)
200–500	0.661	0.242	7.462	**0.006**	1.94(1.21,3.11)
>500	1.613	0.520	9.620	**0.002**	5.02 (1.81,13.92)
Personal willingness to receive the HPV vaccine					
No	reference				
Yes	3.560	0.197	327.057	**<0.001**	35.18(23.92,51.74)
History of vaccine refusal for themselves					
No	reference				
Yes	−0.683	0.241	8.001	**0.005**	0.51(0.32,0.81)

CI, Confidence interval; SE, Standard error. Bold: results significant (*p* < 0.05).

#### 3.1.5 Other facilitators, perspectives, and attitudes of caregivers’ willingness to vaccinate their adolescent daughters against HPV

Regarding whether they and their families should get vaccinations, 59.95% of caregivers preferred their physician’s advice, while a minority favored their own opinions (17.44%) and those of family members (19.11%). A minimal number considered the viewpoints of social media users (6.68%), friends, colleagues, or classmates (1.38%), and public figures (1.00%) to be more significant. In terms of information access, the majority of caregivers expressed a preference for obtaining HPV vaccine information through consultations with physicians (72.92%) or through public lectures at hospitals and schools (57.78%). Nearly half of the caregivers were amenable to receiving information via social public welfare campaigns (44.68%), or through TV or the Internet (40.63%), while 21.28% indicated a willingness to acquire information from relatives or friends. Regarding vaccination sites, 36.59 and 21.23% of caregivers preferred to get HPV vaccination at CDCs or hospitals, respectively. 19.77% preferred school hospitals or schools, while only 4.26% opting for community health service centers. 23.61% of caregivers believed that all four organizations could provide HPV vaccinations to their children. If the government organizes HPV vaccinations, in terms of the choice of vaccination method, nearly half of the caregivers (49.14%) expressed a desire for the government to offer a subsidy, allowing caregivers to voluntarily select the type of vaccine administered. 31.25% of caregivers expressed a willingness to get the government-designated bivalent vaccine at no cost, while 19.15% stated they would consent to vaccinate their children, depending on the government’s provision of the vaccine, regardless of subsidy status. Furthermore, if the government implements a vaccine subsidy program, 85.61% of caregivers indicated that they would be more willing to vaccinate their children against HPV, and only 10.93 and 3.46% expressed uncertainty or reluctance, respectively. See Table 6 for details.

**Table 6:** Other facilitators, perspectives, and attitudes of caregivers’ willingness to vaccinate their adolescent daughters against HPV (*n* = 2,397).

Variables	N	%
Whose opinion do caregivers value more when deciding whether to vaccinate themselves and their family members?		
Doctors	1,437	59.95
Family members	458	19.11
Caregivers themselves	418	17.44
Attitudes on social media about the HPV vaccine	160	6.68
Friends, classmates, or colleagues	33	1.38
Public figures	24	1.00
Caregivers’ preferred access to HPV vaccine information		
Doctor consultation	1748	72.92
Public lectures in hospitals or schools	1,385	57.78
Social public service campaigns	1,071	44.68
TV/ the Internet	974	40.63
Relatives or friends	510	21.28
I do not want to know about the HPV vaccine	48	2.00
Institutions where caregivers are more willing to have their children get the HPV vaccine		
CDC	877	36.59
Hospitals	509	21.23
School hospitals or school-organized vaccination	474	19.77
Community health service center	102	4.26
Any of these options	566	23.61
Caregivers’ attitudes toward the organization method if the government organizes HPV vaccination		
Willing to receive government subsidies and choose vaccines voluntarily	1,178	49.14
The subsidy is not important as long as the government provides the vaccine	749	31.25
Willing to receive the government-designated bivalent vaccine for free	459	19.15
If the government provides a subsidy policy for vaccination, would caregivers be more willing to have their children receive the HPV vaccine?		
Yes	2052	85.61
No	83	3.46
It does not matter	262	10.93

HPV, Human papillomavirus; CDC, Centers for Disease Control and Prevention.

### 3.2 Qualitative study results

The qualitative interviews enrolled a total of 75 participants, comprising 30 vaccination-related personnel and health department leaders and 45 caregivers of girls aged 9–14, equally distributed across the GAL regions. Of these, 22 (29.33%) were males and 53 (70.67%) were females. The perspectives of both respondent groups regarding HPV vaccination underwent thematic framework analysis, structured into four primary dimensions: vaccination status, support, challenges, and recommendations. Relevant keywords were extracted for each dimension (see Table 7).

**Table 7:** The analysis results of the thematic framework.

Main themes	Sub-themes	Information summary
Subjects: Vaccination-related personnel and health department leaders		
Support	Policy support	(1) Ganzi: Medical insurance coverage for Category 2 vaccines (e.g., HPV). (2) Aba: Free bivalent HPV vaccines for girls aged 9–14. (3) Cervical cancer screening is included in government assessment indicators, but HPV vaccination is not.
	Project support	Aba Prefecture: Plans to implement the Tencent-supported “Comprehensive Prevention and Control Demonstration Project for Eliminating Cervical and Breast Cancer in Low-Resource Areas of China.”
Status	Vaccination models	(1) Ganzi/Liangshan: Self-paid vaccination (2) Aba: ①Free bivalent HPV vaccines for girls aged 9–14. ②Self-paid vaccination for women aged 15–45
	Coverage rates	(1) Ganzi/Liangshan: Limited vaccination uptake, with significantly lower coverage in rural/mountainous areas than urban residents. (2) Aba: ①Aged 9–14 cohort: Target population consists of 1,312 girls, with over 400 first doses administered. ②Aged 15–45 cohort: Target population includes 12,554 women, with a coverage rate of 6.43%.
	Willingness	Widespread refusal of bivalent vaccines due to waiting for 9-valent.
	Publicity efforts	(1) Ganzi: Multi-channel campaigns include official WeChat accounts, community grid workers, and parent education workshops, with inadequate Tibetan-language materials in pastoral areas. (2) Aba: School-based publicity achieved higher effectiveness. (3) Liangshan: Limited to verbal explanations at vaccination sites.
Challenges	Supply constraints	(1) Critical lack of quadrivalent/9-valent vaccines. (2) Vaccine cold chain transportation consumes significant human and material resources.
	Insufficient knowledge	(1) Public misconceptions: Preferential waiting for high-valent vaccines. (2) Safety concerns: Concerned about the safety and effectiveness. (3) Professional gaps: Grassroots medical staff unable to explain vaccine differences (e.g., “Can I get 9-valent after bivalent?”)
	Economic barriers	(1) Household-level: 70–80% of rural households cannot afford self-paid vaccines. (2) Government-level: There is no fiscal space for subsidies, and HPV vaccination is excluded from basic public health budgets.
	Accessibility challenges	(1) Geographic: >2-h travel to vaccination sites in pastoral areas. (2) Temporal: Clashes with farming seasons. (3) Service gaps: Understaffed township clinics unable to provide HPV vaccines.
Recommendations	Policy and funding support	(1) Introducing policies of reduced or waived fees for HPV vaccination with financial support from provincial governments. (2) Reimbursing the cost of vaccines through health insurance. (3) Incorporating the HPV vaccine in the national immunization program (NIP).
	Strengthen publicity	(1) Implement diversified health education initiatives, such as producing short videos in Tibetan and Yi languages. (2) Train frontline health workers, focusing on explaining differences in vaccine valency. (3) Conduct science-popularization through school systems, including HPV knowledge transfer sessions during parent-teacher meetings.
	Ensure supply	(1) Expand Service Coverage: Establish additional fixed/mobile vaccination stations, especially in remote and underserved areas. (2) Implement real-time vaccine inventory monitoring at local levels: Develop coordinated allocation systems with provincial CDC centers to prevent shortages.
Subjects: Caregivers of girls aged 9–14		
Status	Coverage rates	(1) Ganzi/Liangshan: The HPV vaccination rate is low (≤10%). (2) Aba: The HPV vaccination rate is high, which benefits from the prefecture government’s free bivalent HPV vaccine policy.
	Considerations	(1) Effectiveness: Whether it can truly and effectively prevent diseases (a primary concern). (2) Safety: Risks of potential short-term and long-term side effects (a core worry). (3) Affordability: Economic affordability is a decisive factor, and free policies have significantly lowered the threshold. (4) The perception that “the higher the valency, the better” is widespread, with a strong preference for the nine-valent vaccine. (5) Trust in Information Sources: Parents have more trust in publicity information from healthcare professionals and school channels.
Challenges	Insufficient knowledge	(1) Most parents, especially those in rural areas of Ganzi and Liangshan, have never heard of the HPV vaccine and lack basic knowledge about it. (2) Misconceptions about vaccine safety exist: Concerns include “potential impact on fertility” and “unknown long-term side effects.” (3) Confusion about valency differences: Some parents only know that “higher valency is better” but do not understand the specific differences in disease protection coverage, and doubts remain about “whether the 9-valent vaccine can be administered after receiving the 2-valent vaccine.” (4) Cognitive bias regarding vaccination age: Beliefs that “children are too young for vaccination” or “vaccination in adulthood is safer.”
	Economic barriers	(1) High cost of self-payment: The total cost of the nine-valent vaccine is about 4,000 RMB, which is far beyond the affordability of low-income families. (2) Uneven policy coverage: There is no universal subsidy policy in Ganzi and Liangshan, and the free policy in Aba is only applicable to the bivalent vaccine. (3) Transportation and work absence costs for traveling to other places for vaccination increase the burden (as mentioned, “it is inconvenient to ask for leave even if the vaccine is obtained”).
	Accessibility challenges	(1) Severe shortage of quadrivalent and nine-valent vaccines: All three regions reported difficulties in making appointments and being unable to get the vaccines, requiring long-term queuing. (2) Limited vaccination sites, scheduling conflicts (e.g., with the agricultural season), and high travel-related.
Recommendations	Policy interventions	(1) Implement a universal free bivalent vaccine policy (referring to the Aba model), with particular coverage of rural/mountainous areas. (2) Expand the scope of subsidies: Provide tiered subsidies to families choosing quadrivalent/nine-valent vaccines.
	Strengthen publicity	(1) Explain the safety of the HPV vaccine through diverse health education initiatives. (2) Clarify differences between vaccine valencies (e.g., the bivalent vaccine can prevent 70% of cervical cancers, while the nine-valent vaccine can prevent approximately 90%). (3) Explain the necessity of early vaccination.
	Ensure supply	(1) Increase the quotas of quadrivalent and nine-valent vaccines in low-resource areas. (2) Establish additional mobile vaccination stations.

#### 3.2.1 Interviews with vaccination-related personnel and health department leaders

In GAL prefectures, the prevention and control of cervical cancer have been prioritized by local governments, with cervical cancer screening being one of the important public health programs. The HPV vaccine is an important mean for cervical cancer prevention and control, but the vaccination status varies across the three regions. In Ganzi and Liangshan Prefectures, vaccination primarily depends on voluntary self-payment, while Aba Prefecture offers free bivalent vaccines to girls aged 9–14 1 month before our survey under specific policies. The local governments in these regions have made some efforts to enhance the knowledge and willingness of residents to get the HPV vaccine through verbal education, television campaigns, WeChat promotions, and printed brochures. However, the coverage of publicity and education is still very limited.

Despite these efforts, HPV vaccination rates for girls in all three regions remained low. In Ganzi and Liangshan Prefectures, the vaccination rate for eligible girls was below 10%. Before the implementation of free HPV vaccination, Aba Prefecture also had a low vaccination rate. Following the initiation of free vaccination this year, the vaccination rate among girls increased rapidly to 30%. The primary challenges in these regions include:

(1) Insufficient vaccine supply and limited vaccination sites: Before 2024, there was a widespread shortage of vaccines across all three regions. Although the supply of the bivalent vaccine has been ensured since 2024, the supply of the quadrivalent and nine-valent vaccines still cannot meet the demand. In these three areas, about 10–20% of caregivers with better economic status would like to vaccinate their girls with quadrivalent and nine-valent vaccines. In addition, some township health centers do not provide HPV vaccination due to insufficient staff. Since the three prefectures of GAL are mountainous regions with poor transportation, township residents who wish to get HPV vaccination must travel long distances to a vaccination site in a neighboring city, which is both time-consuming and inconvenient.
(2) Poor economic conditions: The cost of the HPV vaccine remained unaffordable for most residents, and the local governments’ financial budgets were insufficient to provide vaccination subsidies either.
(3) Limited public knowledge of the HPV vaccine: Most residents lacked the correct knowledge of “the earlier the vaccination, the better the protection,” and they preferred to wait for the nine-valent or quadrivalent vaccines instead of choosing the more readily available bivalent vaccine. Additionally, while some residents were aware that the HPV vaccine could prevent cervical cancer, they lacked sufficient knowledge about the specific differences among different valency types, such as the diseases they prevent and their levels of protection. The majority of remote villages’ residents had never heard of the HPV vaccine and were unfamiliar with HPV-related information.

In summary, although the governments of GAL Prefectures had made active efforts in the promotion of HPV vaccination, they continued to face several obstacles. Overcoming these challenges and difficulties in the future may increase HPV vaccination rates and significantly improve the overall health of women in these regions.

#### 3.2.2 Interviews with caregivers of girls aged 9–14

The results of interviews showed that HPV vaccination rates among girls in Ganzi and Liangshan Prefectures are relatively low, primarily due to caregivers’ limited knowledge about HPV vaccine, difficulties in making vaccination appointments, and poor economic conditions. However, in Aba Prefecture, where the government provided free bivalent HPV vaccines 1 month before our survey, caregivers showed a higher willingness to vaccinate, leading to higher vaccination rates.

Caregivers in all three regions demonstrated insufficient knowledge of the HPV vaccine, and even most in remote rural areas having never even heard of it. Only a minority of caregivers with higher educational levels and better health awareness understood the differences among the bivalent, quadrivalent, and nine-valent vaccines, and tended to vaccinate their children early.

The main reason why caregivers were willing to vaccinate their children was their awareness of the vaccine’s importance in disease prevention. While those unwilling to vaccinate were mainly concerned about the vaccine’s safety and efficacy, as well as financial constraints. Many caregivers indicated that the provision of free vaccines or some financial subsidies by the government, along with enhanced vaccination-related education, would increase their willingness to vaccinate their children.

## 4 Discussion

In recent years, the incidence and mortality rates of cervical cancer in China have shown varying degrees of increase (2), and the burden of HPV-related diseases may be even heavier in areas with scarce health resources, such as the GAL prefectures. The WHO recommends prioritizing girls aged 9–14 who have not yet become sexually active as the primary target group for vaccination, and the immunization strategy should prioritize achieving a high vaccination rate in this population (9). Studies have shown that the ultimate decision regarding children’s HPV vaccination lies with their parents (20). Therefore, understanding the HPV vaccination knowledge among caregivers of school girls in the GAL prefectures and their willingness to vaccinate their children is critical for developing targeted cervical cancer prevention strategies tailored to regions with limited health resources in western China.

Our quantitative study indicated that more than half of the caregivers in GAL prefectures had heard of the HPV virus (59.28) and the HPV vaccines (69.63%), and that caregivers’ willingness to vaccinate girls was relatively strong (92.12%), which was similar to previous national studies in China (75.5, 84.7, and 94.3%) (21, 22). The disparity in public familiarity with HPV and its vaccines may stem from public health initiatives that emphasize the importance of vaccination, rather than adequately educating the public about the virus itself. Health education often focuses on the benefits of the vaccine, especially its role in cancer prevention, and less on the HPV virus. Thus, people tend to be more familiar with the HPV vaccine as a preventive measure than with the virus it targets. Qualitative interviews also showed that caregivers in all three regions generally hold a positive attitude toward HPV vaccination, and most residents had heard of the HPV vaccine. There are several possible explanations. Firstly, most caregivers surveyed were mothers. Many studies have shown that female caregivers of girls are more likely to pay attention to HPV-related information and have a higher willingness to vaccinate (17, 21). Secondly, the National Health Commission has launched a pilot expanded program on HPV immunization for local female adolescents aged 9–14 in selected cities and regions since 2021 (22). The Sichuan provincial government has responded positively and placed significant emphasis on promoting the implementation of HPV vaccination for adolescent females throughout the province (23). The governments of GAL prefectures have also actively publicized and promoted HPV vaccination. Consequently, most caregivers in this study had heard of HPV and its vaccine, and had a high willingness to vaccinate. However, it should be noted that caregivers lacked detailed knowledge about HPV and its vaccine. Only a few caregivers knew that HPV can cause diseases such as anal cancer (14.14%) and genital warts (22.99%), and the majority of respondents were completely unaware of different valency types of HPV vaccine and diseases that can be prevented. This lack of knowledge, as indicated by qualitative findings, may be due to limited education levels among caregivers, economic challenges, and the scarcity of healthcare resources in these regions (21).

Knowledge about HPV and the vaccine may influence caregivers’ willingness to vaccinate their girls. Previous studies pointed out that the primary reason caregivers did not vaccinate their daughters against HPV was a lack of knowledge about HPV vaccination and cervical cancer (24). Conversely, increasing knowledge of HPV vaccines is shown to improve caregivers’ acceptance of vaccination for adolescents (25). Our findings were similar to previous reports (24), with caregivers who were willing to vaccinate their children having significantly higher knowledge scores than those who were unwilling. The interview results also indicated that inadequate knowledge among caregivers was one of the main obstacles to the advancement of HPV immunization in GAL areas. Moreover, our study revealed that, amid the ongoing HPV vaccine shortage, the majority of caregivers preferred immunizing their daughters with the nine-valent vaccine, while only a minority opting for the bivalent or quadrivalent HPV vaccine at the earliest opportunity, which is consistent with data from China (26, 27). Qualitative interview results also indicated that most caregivers had a limited understanding of the concept that “earlier vaccination provides better protection,” and they hold misconceptions regarding the selection of HPV vaccine types, frequently waiting for the higher-valent vaccine for girls to be vaccinated instead of receiving the adequately supplied bivalent vaccine at the appropriate age (28). Consequently, enhancing caregivers’ awareness and knowledge of the HPV vaccination is crucial in encouraging them to vaccinate their daughters against HPV.

The price of HPV vaccines could influence caregivers’ willingness to vaccinate adolescents with HPV vaccine (24, 28). Our study found that caregivers who were willing to pay for vaccination were more willing to vaccinate girls against HPV. However, HPV vaccines often use a three-dose vaccination strategy, and the total price ranges from 1,740 RMB to 3,954 RMB (US$254 to $576) (11). Qualitative findings consistently emphasized that the high cost of vaccination was a major barrier to vaccine promotion. The questionnaire survey also found that nearly 60% of caregivers were either unwilling to pay or simply prepared to pay less than 200 RMB for HPV vaccination, and only 9.26% of caregivers could afford more than 500 RMB, which is similar to the results of previous studies in China (17, 24).

Prior vaccination behaviors among caregivers may influence their willingness to vaccinate their girls with HPV vaccines, which has also been demonstrated in many other studies (21). Our study showed that caregivers who had never refused vaccination or were personally willing to receive the HPV vaccine were more likely to vaccinate their children against HPV. In addition, although only 69.63% of caregivers had heard of the HPV vaccine, 92.12% stillexpressed a willingness to vaccinate their girls. This discrepancy suggests that some caregivers, despite not knowing about the HPV vaccine specifically, are still willing to vaccinate their children. Such caregivers may be influenced by previous vaccination practices, recognize the general importance of vaccines to prevent diseases, and remain open to disease-specific vaccines (29). In contrast, the core concerns of caregivers who refused to vaccinate their children were the safety and efficacy of vaccines. The reasons for refusal, such as “Concerns about potential side effects (28.04%)” and “Uncertainty about the vaccine’s effectiveness (18.52%)” ranked second and third respectively, accounting for about half of the total reasons. These findings reflected caregivers’ doubts about vaccine safety and efficacy, which were consistent with previous literature (11, 30). In qualitative interviews with caregivers, those who were unwilling to vaccinate also expressed major concerns about the safety and efficacy of the vaccine. Notably, social media platforms in rural areas (e.g., TikTok and WeChat) have become significant channels for disseminating misinformation (31). During interviews, caregivers reported exposure to rumors such as “HPV vaccines cause infertility.” These misinformations amplify safety concerns and erode trust, particularly among populations with limited ability to discern information accuracy.

The study also found that most caregivers preferred to follow physician recommendations when deciding on vaccination for themselves and their family members, and they were also more inclined to obtain HPV vaccine information from doctors. Moreover, over half of the caregivers favored vaccination of the girls by the CDC or hospitals. This is supported by previous studies showing that caregivers possess greater trust in their physicians, and a strong recommendation from a healthcare provider can substantiate the need for the HPV vaccine and mitigate caregivers’ vaccine hesitation (20). FGDs with vaccination-related personnel and health department leaders also indicated that training grassroots health workers to deliver clear, evidence-based communication, such as emphasizing that “HPV vaccines prevent cervical cancer as effectively as hepatitis B vaccines prevent liver diseases.” serves as a pivotal strategy for building trust and countering misinformation, which can help to increase vaccination rates. In addition, our study indicated that overall, caregivers preferred either fixed subsidy with free choices of vaccine type or free government-designated bivalent vaccine to vaccinate their daughters. If the government instituted a vaccination subsidy program, it would significantly increase their willingness to vaccinate their daughters against HPV. This aligns with the results of the study in Guangdong Province (28). The success of Aba Prefecture also exemplifies this. Therefore, policymakers may need to consider introducing supportive policies that allow self-selection of HPV vaccine type with subsidies or directly offer free but designated HPV vaccine to enhance HPV vaccination rates among girls of appropriate age.

Caregivers’ willingness to vaccinate girls against HPV was remarkably high in our study. In contrast, HPV vaccination uptake among girls remains notably low in GAL prefectures. Multiple studies have shown a similar discrepancy between vaccination willingness and behavior (32, 33). Qualitative interviews indicated that limited vaccine supply, high costs, limited accessibility, and public concerns about safety and efficacy are major barriers impacting vaccination rates, which were similar to the findings of previous studies (32, 34). It is worth noting that potential cultural and contextual barriers were identified in GAL prefectures, where Tibetan and Yi ethnic communities were concentrated. Firstly, these ethnic groups had their distinct languages, which hindered the effective delivery of health education initiatives. Secondly, the Tibetan and Yi populations primarily relied on farming, and the timing of agricultural activities frequently conflicted with scheduled vaccinations at health centers, making it difficult for caregivers to allocate time to accompany their daughters for vaccinations. This discrepancy underscores that willingness alone is insufficient. Policymakers must address both structural barriers, including availability and accessibility, and cognitive barriers, to translate willingness to vaccinate into actual action.

The implementation of the “Free Bivalent HPV Vaccination for Girls Aged 9–14” policy in Aba Prefecture substantially raised caregivers’ willingness to vaccinate their children and significantly elevated local HPV vaccination rates. This experience provides a valuable replicable model. While financial subsidies or free vaccination are necessary, they must be complemented by ensuring vaccine accessibility and effective health education delivered by healthcare workers to ultimately maximize coverage rates. Based on our findings, we recommend several strategies to increase caregivers’ willingness to vaccinate their eligible girls against HPV. Firstly, diverse payment options should be developed to make the HPV vaccine more affordable. Health policymakers and governments need to explore feasible approaches to alleviate economic constraints, such as introducing policies of reduced or waived fees for HPV vaccination with financial support from provincial governments, reimbursing the cost of vaccines through health insurance, or incorporating the HPV vaccine in the national immunization program (NIP) to ensure stable vaccine supply and achieve high coverage rates. Secondly, it is essential to improve access to vaccination services. It is recommended that more vaccination points be established or that mobile vaccination stations be set up to cover more areas, particularly in remote and underserved areas. Furthermore, ensuring a sufficient supply of vaccines is crucial to prevent shortages that could disrupt vaccination programs. Local governments should take the lead in establishing a vaccine supply assurance mechanism, including real-time monitoring of regional vaccine inventories and coordinated allocation systems with provincial CDC centers, to maintain a stable and continuous vaccine supply. Thirdly, awareness of HPV/HPV vaccines among targeted caregivers should be increased to reduce their concerns about the safety and efficacy of the vaccine. Studies have confirmed that education is the most effective way to improve the knowledge level, ease concerns about safety and effectiveness, and improve the acceptability of the HPV vaccine of the people (17, 24). Therefore, healthcare providers can deliver targeted educational interventions that emphasize the safety and necessity of the HPV vaccine (35), and make full use of public media (such as TV, newspapers, and the internet) to disseminate information. It is important to note that public health messages directed at caregivers of adolescent girls should avoid overemphasizing the numerical difference in vaccine coverage (i.e., the number of HPV viruses covered) (23). Instead, they should clearly communicate that all vaccine types are effective against high-risk HPV and emphasize that available vaccine products in the market are equally acceptable (23). These measures could facilitate the widespread availability of the HPV vaccine.

This study has several limitations. First, all data were self-reported, and some caregivers might have chosen not to answer the entire survey or skipped specific questions within it. This non-random missing data could potentially lead to underestimation or overestimation of parameters in our study. However, we excluded incomplete questionnaires from our analysis, suggesting that the results based on the current analysis may still be reliable. Second, there might be potential response bias, as respondents could have been inclined to provide socially desirable answers. Third, the population included a higher proportion of mothers, which could reflect some degree of participant bias. However, whether to vaccinate girls against HPV is more often decided by their mothers. Despite these limitations, this study’s principal strength lies in its focus on the GAL prefectures in western China, where healthcare resources are scarce. To our knowledge, this represents the first mixed-methods assessment of HPV vaccine acceptability in GAL, uniquely evaluating the impact of localized policies (e.g., free vaccination in Aba Prefecture) on uptake. By integrating socioeconomic barriers and health system challenges into our analysis, we provide actionable evidence for designing equity-focused HPV vaccination policies in resource-limited areas of western China.

## 5 Conclusion

In conclusion, although HPV vaccination rates among primary and secondary school girls in GAL prefectures remains low, caregivers generally hold a positive attitude toward vaccination. Vaccination rates are largely influenced by government policies, economic conditions, caregivers’ knowledge, and vaccine accessibility. To address these challenges, policy efforts should prioritize developing diverse payment options to alleviate financial burdens, ensuring a stable and sufficient vaccine supply, and enhancing caregivers’ awareness through targeted education campaigns using trusted physicians and local dialects. Moreover, increasing vaccination accessibility by setting up more vaccination sites or mobile units in underserved areas could help reduce regional disparities. These findings provide valuable evidence for developing effective HPV vaccination strategies in resource-limited regions of western China.

## Data Availability

The raw data supporting the conclusions of this article will be made available by the authors, without undue reservation.

## References

[1] Chan CK Aimagambetova G Ukybassova T Kongrtay K Azizan A. Human papillomavirus infection and cervical Cancer: epidemiology, screening, and vaccination-review of current perspectives. J Oncol. (2019) 2019:3257939. doi: 10.1155/2019/3257939, PMID: 31687023 PMC6811952

[2] de Martel C Georges D Bray F Ferlay J Clifford GM. Global burden of cancer attributable to infections in 2018: a worldwide incidence analysis. Lancet Glob Health. (2020) 8:e180–90. doi: 10.1016/s2214-109x(19)30488-7, PMID: 31862245

[3] Guo Q Luo K Hu R. The spatial correlations of health resource agglomeration capacities and their influencing factors: evidence from China. Int J Environ Res Public Health. (2020) 17:8705. doi: 10.3390/ijerph17228705, PMID: 33238597 PMC7700579

[4] Luo Q Jiang N Wu Q Wang J Zhong J. Prevalence and genotype distribution of HPV and cervical pathological results in Sichuan Province, China: a three years surveys prior to mass HPV vaccination. Virol J. (2020) 17:100. doi: 10.1186/s12985-020-01366-2, PMID: 32650791 PMC7350733

[5] Luo LP He P Liu QT Jiang YH Zhang YN Li QZ. Prevalence and genotype distribution of HPV infection among 214,715 women from southern China, 2012-2018: baseline measures prior to mass HPV vaccination. BMC Infect Dis. (2021) 21:328. doi: 10.1186/s12879-021-06019-5, PMID: 33827456 PMC8028771

[6] Ding L Zhang N Mao Y. Addressing the maldistribution of health resources in Sichuan Province, China: a county-level analysis. PLoS One. (2021) 16:e0250526. doi: 10.1371/journal.pone.0250526, PMID: 33891649 PMC8064550

[7] Zhou M. Equity and efficiency of health resource allocation in township health centers in Sichuan Province, China. PLoS One. (2024) 19:e0299988. doi: 10.1371/journal.pone.0299988, PMID: 38442112 PMC10914297

[8] Brisson M Kim JJ Canfell K Drolet M Gingras G Burger EA. Impact of HPV vaccination and cervical screening on cervical cancer elimination: a comparative modelling analysis in 78 low-income and lower-middle-income countries. Lancet. (2020) 395:575–90. doi: 10.1016/s0140-6736(20)30068-4, PMID: 32007141 PMC7043009

[9] Smolarczyk K Duszewska A Drozd S Majewski S. Parents’ knowledge and attitude towards HPV and HPV vaccination in Poland. Vaccines (Basel). (2022) 10:228. doi: 10.3390/vaccines10020228, PMID: 35214686 PMC8876926

[10] Wu D Liu P Song D Wang H Chen S Tang W. Implementing the free HPV vaccination for adolescent girls aged below 14 in Shenzhen, Guangdong Province of China: experience, challenges, and lessons. Infect Dis Poverty. (2023) 12:98. doi: 10.1186/s40249-023-01149-1, PMID: 37899444 PMC10614323

[11] Zhao F Qiao Y. Cervical cancer prevention in China: a key to cancer control. Lancet. (2019) 393:969–70. doi: 10.1016/s0140-6736(18)32849-6, PMID: 30860036

[12] Staples JN Nelamangala SL Morris S Wells K. Exploring socio-demographic and geospatial variation in human papillomavirus vaccination uptake in Virginia. Vaccine. (2021) 39:5385–90. doi: 10.1016/j.vaccine.2021.07.079, PMID: 34384637

[13] Bruni L Diaz M Barrionuevo-Rosas L Herrero R Bray F Bosch FX. Global estimates of human papillomavirus vaccination coverage by region and income level: a pooled analysis. Lancet Glob Health. (2016) 4:e453–63. doi: 10.1016/s2214-109x(16)30099-7, PMID: 27340003

[14] Jin SW Lee Y Brandt HM. Human papillomavirus (HPV) vaccination knowledge, beliefs, and hesitancy associated with stages of parental readiness for adolescent HPV vaccination: implications for HPV vaccination promotion. Trop Med Infect Dis. (2023) 8:251. doi: 10.3390/tropicalmed8050251, PMID: 37235299 PMC10222878

[15] Galanis P Vraka I Siskou O Konstantakopoulou O Katsiroumpa A Kaitelidou D. Willingness, refusal and influential factors of parents to vaccinate their children against the COVID-19: a systematic review and meta-analysis. Prev Med. (2022) 157:106994. doi: 10.1016/j.ypmed.2022.106994, PMID: 35183597 PMC8861629

[16] Min Z Lili G Hongying Y. Cognition investigation of female patients to cervical cancer screening and HPV vaccine in Chengdu area. Chongqing Med. (2018) 47:786–8.

[17] Yu Y Xu M Sun J Li R Li M Wang J. Human papillomavirus infection and vaccination: awareness and knowledge of HPV and acceptability of HPV vaccine among mothers of teenage daughters in Weihai, Shandong, China. PLoS One. (2016) 11:e0146741. doi: 10.1371/journal.pone.0146741, PMID: 26766565 PMC4713086

[18] Jaspan DM Dunton CJ Cook TL. Acceptance of human papillomavirus vaccine by gynecologists in an urban setting. J Low Genit Tract Dis. (2008) 12:118–21. doi: 10.1097/LGT.0b013e31815d9639, PMID: 18369305

[19] Netfa F King C Davies C Rashid H Tashani M Booy R. Knowledge, attitudes, and perceptions of the Arabic-speaking Community in Sydney, Australia, toward the human papillomavirus (HPV) vaccination program: a qualitative study. Vaccines (Basel). (2021) 9:940. doi: 10.3390/vaccines9090940, PMID: 34579177 PMC8473026

[20] Sonawane K Zhu Y Montealegre JR Lairson DR Bauer C McGee LU. Parental intent to initiate and complete the human papillomavirus vaccine series in the USA: a nationwide, cross-sectional survey. Lancet Public Health. (2020) 5:e484–92. doi: 10.1016/s2468-2667(20)30139-0, PMID: 32707126 PMC7484349

[21] Xie H Zhu HY Jiang NJ Yin YN. Awareness of HPV and HPV vaccines, acceptance to vaccination and its influence factors among parents of adolescents 9 to 18 years of age in China: a cross-sectional study. J Pediatr Nurs. (2023) 71:73–8. doi: 10.1016/j.pedn.2023.03.007, PMID: 37028228

[22] Yi Y Xiu S Shi N Huang Y Zhang S Wang Q. Perceptions and acceptability of HPV vaccination among parents of female adolescents 9-14 in China: a cross-sectional survey based on the theory of planned behavior. Hum Vaccin Immunother. (2023) 19:2225994. doi: 10.1080/21645515.2023.2225994, PMID: 37340698 PMC10332177

[23] Yim VW Wang Q Li Y Qin C Tang W Tang S. Between now and later: a mixed methods study of HPV vaccination delay among Chinese caregivers in urban Chengdu, China. BMC Public Health. (2024) 24:183. doi: 10.1186/s12889-024-17697-6, PMID: 38225563 PMC10790461

[24] Wang W Ma Y Wang X Zou H Zhao F Wang S. Acceptability of human papillomavirus vaccine among parents of junior middle school students in Jinan, China. Vaccine. (2015) 33:2570–6. doi: 10.1016/j.vaccine.2015.04.010, PMID: 25887088

[25] Lindsay AC Pineda JA Valdez MJ Torres MI Granberry PJ. Central American immigrant parents’ awareness, acceptability, and willingness to vaccinate their adolescent children against human papillomavirus: a pilot cross-sectional study. Int J Environ Res Public Health. (2020) 17:2869. doi: 10.3390/ijerph17082869, PMID: 32326320 PMC7215825

[26] Wei Z Liu Y Zhang L Sun X Jiang Q Li Z. Stages of HPV vaccine hesitancy among guardians of female secondary school students in China. J Adolesc Health. (2023) 72:73–9. doi: 10.1016/j.jadohealth.2022.08.027, PMID: 36229401 PMC9746349

[27] Lin Y Su Z Chen F Zhao Q Zimet GD Alias H. Chinese mothers’ intention to vaccinate daughters against human papillomavirus (HPV), and their vaccine preferences: a study in Fujian Province. Hum Vaccin Immunother. (2021) 17:304–15. doi: 10.1080/21645515.2020.1756152, PMID: 32401617 PMC7872083

[28] Xie P Zhao J Li X Zou X Liu G Han X. Preference for human papillomavirus vaccine type and vaccination strategy among parents of school-age girls in Guangdong province, China. Prev Med Rep. (2023) 36:102463. doi: 10.1016/j.pmedr.2023.102463, PMID: 37854667 PMC10580040

[29] Zhang SK Pan XF Wang SM Yang CX Gao XH Wang ZZ. Perceptions and acceptability of HPV vaccination among parents of young adolescents: a multicenter national survey in China. Vaccine. (2013) 31:3244–9. doi: 10.1016/j.vaccine.2013.05.046, PMID: 23707446

[30] Lane S MacDonald NE Marti M Dumolard L. Vaccine hesitancy around the globe: analysis of three years of WHO/UNICEF joint reporting form data-2015-2017. Vaccine. (2018) 36:3861–7. doi: 10.1016/j.vaccine.2018.03.063, PMID: 29605516 PMC5999354

[31] Loeb S Langford AT Bragg MA Sherman R Chan JM. Cancer misinformation on social media. CA Cancer J Clin. (2024) 74:453–64. doi: 10.3322/caac.21857, PMID: 38896503 PMC11648589

[32] Guo Q Zhou W Wen X Lu J Lu X Lu Y. Discrepancy of human papillomavirus vaccine uptake and intent between girls 9-14 and their mothers in a pilot region of Shanghai, China. Hum Vaccin Immunother. (2022) 18:2132801. doi: 10.1080/21645515.2022.2132801, PMID: 36306482 PMC9746362

[33] Ye L Fang T Cui J Zhu G Ma R Sun Y. The intentions to get vaccinated against influenza and actual vaccine uptake among diabetic patients in Ningbo, China: identifying motivators and barriers. Hum Vaccin Immunother. (2021) 17:106–18. doi: 10.1080/21645515.2020.1761201, PMID: 32460620 PMC7877400

[34] Do EK Rossi B Miller CA Ksinan AJ Wheeler DC Chukmaitov A. Area-level variation and human papillomavirus vaccination among adolescents and young adults in the United States: a systematic review. Cancer Epidemiol Biomarkers Prev. (2021) 30:13–21. doi: 10.1158/1055-9965.Epi-20-0617, PMID: 33008874 PMC8108385

[35] Vasudevan L Ostermann J Wang Y Harrison SE Yelverton V Fish LJ. Association of caregiver attitudes with adolescent HPV vaccination in 13 southern US states. Vaccine X. (2022) 11:100181. doi: 10.1016/j.jvacx.2022.100181, PMID: 35789674 PMC9250032

