# Supplementary material for: Barriers and enablers of HPV vaccination among caregivers of school-age girls in resource-limited regions of Western Sichuan, China: a mixed-methods study
Source: Front Public Health. 2025 Aug 20;13:1618935. doi: 10.3389/fpubh.2025.1618935 (PMC12405240; doi:10.3389/fpubh.2025.1618935)
Supplement: Supplementary file 1 [file Supplementary_file_1.docx]

**Appendix A. Survey on Vaccination Willingness of Caregivers**

| Dear Caregiver,  You are invited to participate in a survey that will contribute valuable information for developing disease prevention and control strategies in our country. Thank you for your cooperation!  I agree to participate in this survey. Caregiver's Signature___________  Instructions for Completing the Form: Please mark '√' next to the option that applies. Unless indicated as 'multiple choice,' each question is single choice only.  (This survey refers to children under the age of 15 in the household, primarily targeting girls in the household.)  **Part 1: Basic Information ID Number：________**  **A1. Caregiver’s Birth Date：**\|__\|__\|__\|__\|-\|__\|__\|-\|__\|__\|  **A1.1 Relationship of Caregiver to the Child:**  ①Mother ② Father ③ Paternal Grandmother  ④ Maternal Grandmother ⑤ Paternal Grandfather  ⑥ Maternal Grandfather  ⑦ Other, please specify__________  **A2. Caregiver's Gender:**  ① Male ② Female  **A2.1 Does the household have a girl under the age of 15?**  ① Yes ② No  **A3. Is the child the only child?**  ① Yes ② No  **A3.1 School the child attends:**  ①Secondary School, School Name______  ② Primary School, School Name______  **A4. Residence:**  ① Rural ② Urban  **A4.1 Region:**  ① Ganzi Prefecture  ② Aba Prefecture  ③ Liangshan Prefecture  **A5. Ethnicity:**  ① Han ② Tibetan ③ Yi ④ Qiang  ⑤ Other, please specify_______  **A6. Caregiver's Education Level:**  ① No Formal Education  ② Primary School |  | ③ Junior High School ④ Senior High School  ⑤ College or Above  **A7. Caregiver's Occupation:**  ① Staff/Officer ② Factory Worker/Industrial Worker  ③ Service Worker ④ Businessperson/Professional  ⑤ Housewife/ Homemaker  ⑥ Other, please specify__________  **A8. Caregiver's Marital Status:**  ① Unmarried ② Married ③ Divorced ④ Widowed  **A8.1 Marital Status of the Child's Parents:**  ①Unmarried ② Married ③ Divorced ④ Widowed  **Part 2: Knowledge and Attitudes Towards HPV Vaccine**  **B1. Have you heard of Human Papillomavirus (HPV)?**  ① Yes ② No  **B2. Have you heard of the HPV vaccine?**  ① Yes ② No  **B3. When it comes to deciding whether to vaccinate yourself or your family, whose opinion do you value most?[Select all that apply]**  ① Your own ② Family members ③ Doctors  ④ Friends, classmates, or colleagues  ⑤ Attitudes on social media about the HPV vaccine  ⑥ Public figures  **B4. Which of the following diseases can the HPV vaccine effectively prevent? [Select all that apply]**  ① Cervical cancer ② Anal cancer ③ Genital warts  ④ Ovarian cancer ⑤ I don't know  **B5. Through which channels do you prefer to receive information about the HPV vaccine? [Select all that apply]**  ① Doctor consultation ② Relatives or friends  ③ Public lectures at hospitals/schools  ④ Social public service campaigns  ⑤ TV/Internet  ⑥ I don't want to know about HPV vaccine.  **B6. Have doctors recommended the HPV vaccine to you or your family?**  ① Yes ② No |
| --- | --- | --- |
| **B7. If given the opportunity, which type of HPV vaccine would you or your family be willing to receive?**  ① Bivalent (skip to B9) ② Quadrivalent (skip to B9)  ③ Nine-valent (skip to B9)  ④ I don't know the differences between these types (skip to B9)  ⑤ I am not willing to get vaccinated  **B8. What concerns do you have about receiving or recommending the HPV vaccine for yourself or your family? [Select all that apply]**  ① Insufficient knowledge about the vaccine  ② Concerns about potential side effects  ③ High cost of the vaccine  ④ Uncertainty about the vaccine's effectiveness  ⑤ Consider that there is no risk of cervical cancer  ⑥ Consider that the vaccine has not been widely promoted  ⑦ Concerns about counterfeit or expired vaccines  ⑧ No professional recommendation  ⑨ No nearby HPV vaccination sites  ⑩ Insufficient time for vaccination  ⑪ Difficult appointment for the HPV vaccine  **B9. If given the opportunity, which type of HPV vaccine would you prefer for your child?**  ① I do not have a girl (skip to B12)  ② Bivalent (skip to B11)  ③ Quadrivalent (skip to B11)  ④ Nine-valent (skip to B11)  ⑤ I don't know the differences between these types (skip to B11)  ⑥ Let the girl decide herself (skip to B11)  ⑦ Not willing to vaccinate  **B10. What are your reasons for not wanting your girl to receive the HPV vaccine? [Select all that apply]**  ① Insufficient knowledge about the vaccine  ② Concerns about potential side effects  ③ High cost of the vaccine  ④ Uncertainty about the vaccine's effectiveness  ⑤ Consider that their daughter or granddaughter is not at risk for cervical cancer  ⑥ Consider that the vaccine has not been widely promoted |  | ⑦ Concerns about counterfeit or expired vaccines  ⑧ No professional recommendation  ⑨ No nearby HPV vaccination sites  ⑩ Insufficient time for HPV vaccination  ⑪ Difficult appointment for the HPV vaccine  ⑫ Concerns that the vaccine might promote premature sexual activity in young girls  ⑬ No recommendation from friends or family for junior high school girls  **B11. What are your reasons for wanting your girl to receive the HPV vaccine?**  ① Strongly recommended by physicians  ② The cost of vaccine is within an acceptable range  ③ Family history of cervical disease  ④ Strongly recommended by family  ⑤ The majority of friends are willing to get vaccinated  ⑥ Vaccination is the most important means of preventing cervical cancer  ⑦ The safety of the vaccine is assured  **B12. Are you willing to pay a portion of the cost for the vaccine?**  ① No (please skip to B12.2)  ② Yes (please answer B12.1)  **B12.1 If you need to pay a portion of the cost each time, what is the maximum amount you are willing to pay?**  ① Less than or equal to 100 RMB  ② 100–200 RMB  ③ 200–300 RMB  ④ 300–400 RMB  ⑤ 400–500 RMB  ⑥ More than 500 RMB  **B12.2 If you are not willing, what are your reasons? [Select all that apply]**  ① High cost  ② Unnecessary  ③ Financial difficulties  ④ Other, please specify __________  **B13. What do you think is the best age range for receiving the HPV vaccine?**  ① Before 15 years old  ② 16–26 years old  ③ 27–45 years old  ④ Any age is fine ⑤ I don’t know |
| **B14. Do you believe the vaccine can protect you and your family from cervical diseases?**  ① Yes ② No  **B15. Will you receive all the vaccines included in the immunization schedule and those recommended by the country?**  ① Yes ② No  **B16. Have you ever intentionally delayed or hesitated to receive a vaccine?**  ① Yes ② No  **B17. Have you ever refused to receive a vaccine?**  ① Yes  ② No  **B18. Will you ensure your child receives all vaccines included in the immunization schedule and those recommended by the country?**  ① Yes ② No  **B19. Have you ever intentionally delayed or hesitated to vaccinate your child?**  ① Yes ② No  **B20. Have you ever refused to vaccinate your child?**  ① Yes ② No  **B21. Has your child received any self-paid vaccines?**  ① Yes ② No  **B22. Has your child been vaccinated with the HPV vaccine?**  ① Yes, please specify the type of vaccine received ______  ② No (skip to B23)  ③ There is no girl in the family (skip to B23)  **B22.1 The date your child received the HPV vaccine** \|__\|__\|__\|__\|-\|__\|__\|  **B22.2 The reason your child received the HPV vaccine**  ① It was organized by the school as a free or subsidized vaccination  ② It was a self-paid vaccination appointment, unrelated to any program  ③ Other, please specify __________  **B23. Given the current shortage of HPV vaccines in China, would you prefer your child to receive the bivalent or quadrivalent vaccine as soon as possible or wait for the nonavalent HPV vaccine?** |  | ① Wait for the nine-valent HPV vaccines  ② Receive the bivalent or quadrivalent vaccine as early as possible  ③ Not sure  ④ Refuse HPV vaccination  **B24. When choosing the type of HPV vaccine for your child, which factors are most important to you? Please rank the importance of the factors by assigning numbers 1, 2, 3, 4, and 5, where 1 represents the most important.**  ① The effectiveness of the vaccine in preventing diseases ___  ② The price of the vaccine ______  ③ The safety of the vaccine ______  ④ Recommendations from teachers or the school____  ⑤ Government organization ______  **B25. Where would you prefer your child to receive the HPV vaccine? [Select all that apply]**  ① CDC (Center for Disease Control)  ② Community health service center  ③ School hospitals or school-organized vaccination  ④ Hospitals  ⑤ Any of these options  **B26. Would you prefer to participate in a vaccination program organized by the school or community?**  ① Yes ② No ③ It doesn’t matter  **B27. If the government provided a subsidy policy for vaccinations, would you be more willing to have your child vaccinated with the HPV vaccine?**  ① Yes ② No ③ It doesn’t matter  **B28. If the government organizes HPV vaccination, what is your attitude towards the organization method? [Select all that apply]**  ① Willing to receive government subsidies and choose vaccines voluntarily  ② Prefer free vaccination with the government-specified bivalent vaccine  ③ As long as the government provides the vaccine, the subsidy is not important  **End of the survey. Thank you for your participation and best wishes for your health!**  **Surveyor's Signature: ________** |

**Appendix B. Interview Outline for Factors Influencing Caregivers' Willingness to Vaccinate Adolescent Daughters with HPV Vaccine in Western China's Impoverished Areas**

**Dear Leaders and Healthcare Personnel:**

You are invited to participate in an interview for the Merck Investigator Studies Program (MISP) titled “Cross-sectional Study on Factors Influencing Guardians' Willingness to Vaccinate Adolescent Daughters with HPV Vaccine in Western China's Impoverished Areas,” conducted by Sichuan University West China School of Public Health and West China Fourth Hospital. This study aims to investigate the awareness, willingness, and influencing factors related to the HPV vaccine in the Ganan-Liang region of western China, which faces poverty and limited healthcare accessibility. We will also analyze the impact of health programs and policies on HPV vaccination rates and vaccine selection.

1. Is cervical cancer considered a significant disease for prevention in the local area? Are there any cervical cancer screening and HPV vaccination programs in place?

2. What methods are used for the administration of the HPV vaccine locally? Are there any programs or policy supports for it?

3. Has there been any public awareness campaign about the HPV vaccine? What are the main methods, channels, or platforms used for this promotion?

4. What is the current situation regarding the HPV vaccination among women in the area? What are the reasons affecting HPV vaccine uptake?

5. How is the situation regarding the vaccination of Category II vaccines in the area? What are the typical and highest prices for these vaccines? What is the public attitude towards receiving Category II vaccines?

6. At vaccination sites or during the vaccination process, do the institutions or doctors provide information about Category II vaccines, or are there any displays or screens promoting related knowledge?

7. What is the supply situation of the HPV vaccine locally? How aware are people of the HPV vaccine and cervical cancer?

8. Does the local government support the promotion of HPV vaccination? Is the government willing to organize implementation, and what forms would they consider adopting?

9. If HPV vaccination is self-funded, is the local population's economic status sufficient to cover the costs? Could the government potentially implement subsidy programs similar to those in Chengdu? What challenges does the government face in organizing such programs, and what solutions might be proposed?

10. Are local policymakers consulting experts in relevant fields when formulating vaccination-related policies?

11. In your opinion, what obstacles and challenges might arise when promoting HPV vaccination in impoverished areas, and how can they be addressed?

**Appendix C. Interview Guide on Factors Influencing Caregivers' Willingness to Vaccinate Adolescent Girls with HPV Vaccine in Poor Areas of Western China — For Students’ Caregivers**

**Introduction:**

**Cervical cancer is a malignant tumor that poses a serious threat to women's health and has caused a significant disease burden worldwide, especially in developing countries. In 2020, the World Health Organization (WHO) launched the Global Strategy to Accelerate the Elimination of Cervical Cancer, outlining the "90-70-90" interim goals for its eradication. Currently, countries and regions around the world are taking action to achieve this shared goal. Cervical cancer screening and preventive HPV vaccination play a crucial role in this effort, significantly reducing the burden of the disease.**

**We are very grateful that you are willing to participate in our interview. During the interview, we will ask you to share your attitudes toward HPV vaccination, and we also hope you can provide valuable suggestions for improving HPV vaccination rates. Additionally, we may need to record and take notes during the interview to ensure that we capture everything you say. If you prefer not to have the interview recorded or wish to stop the recording at any point, please feel free to let us know. If everything is clear, we can begin the interview.**

1. Are there many girls in your child's class or surrounding area who have received the HPV vaccine? Why?

2. What do you think are the reasons parents choose to vaccinate their children with the HPV vaccine? What are the reasons for not vaccinating?

3. What do you think are the main factors parents consider when choosing a vaccine for their children?

4. How knowledgeable are the parents in your child's class or surrounding area about the HPV vaccine? Do they know the differences between the bivalent, quadrivalent, and nine-valent vaccines? What considerations do they have when choosing the type of vaccine?

5. Do you think the girls who haven't been vaccinated yet will receive the HPV vaccine in the next few years? Why or why not?

6. Are you aware of the current government policies regarding HPV vaccination in our country?

7. In 2021, Chengdu implemented a policy where the government provides a fixed subsidy and individuals voluntarily choose the HPV vaccine. Do you think similar subsidy policies would influence parents' willingness to vaccinate their children and their choice of vaccine? How significant would the impact be? What type of organizational structure would parents prefer?

8. What do you think is the biggest obstacle to implementing HPV vaccination programs in your area? Do you have any suggestions for overcoming these challenges?

**Closing Remarks:**

**This concludes the interview. Thank you for your cooperation and valuable suggestions. I wish you a happy and healthy life!**
